# Glucagon-like peptide-1 receptor agonists and sodium-glucose cotransporter 2 inhibitors, anti-diabetic drugs in heart failure and cognitive impairment: potential mechanisms of the protective effects

**DOI:** 10.3389/fphar.2024.1422740

**Published:** 2024-06-14

**Authors:** Maria Antonietta Riemma, Elena Mele, Maria Donniacuo, Marialucia Telesca, Gabriella Bellocchio, Giuseppe Castaldo, Francesco Rossi, Antonella De Angelis, Donato Cappetta, Konrad Urbanek, Liberato Berrino

**Affiliations:** ^1^ Department of Experimental Medicine, University of Campania “Luigi Vanvitelli”, Naples, Italy; ^2^ Department of Biological and Environmental Sciences and Technologies, University of Salento, Lecce, Italy; ^3^ Department of Molecular Medicine and Medical Biotechnologies, University of Naples “Federico II”, Naples, Italy; ^4^ CEINGE-Advanced Biotechnologies, Naples, Italy

**Keywords:** heart failure, cognitive impairment, type 2 diabetes, glucagon-like peptide-1 receptor agonists, sodium-glucose cotransporter 2 inhibitors

## Abstract

Heart failure and cognitive impairment emerge as public health problems that need to be addressed due to the aging global population. The conditions that often coexist are strongly related to advancing age and multimorbidity. Epidemiological evidence indicates that cardiovascular disease and neurodegenerative processes shares similar aspects, in term of prevalence, age distribution, and mortality. Type 2 diabetes increasingly represents a risk factor associated not only to cardiometabolic pathologies but also to neurological conditions. The pathophysiological features of type 2 diabetes and its metabolic complications (hyperglycemia, hyperinsulinemia, and insulin resistance) play a crucial role in the development and progression of both heart failure and cognitive dysfunction. This connection has opened to a potential new strategy, in which new classes of anti-diabetic medications, such as glucagon-like peptide-1 receptor (GLP-1R) agonists and sodium-glucose cotransporter 2 (SGLT2) inhibitors, are able to reduce the overall risk of cardiovascular events and neuronal damage, showing additional protective effects beyond glycemic control. The pleiotropic effects of GLP-1R agonists and SGLT2 inhibitors have been extensively investigated. They exert direct and indirect cardioprotective and neuroprotective actions, by reducing inflammation, oxidative stress, ions overload, and restoring insulin signaling. Nonetheless, the specificity of pathways and their contribution has not been fully elucidated, and this underlines the urgency for more comprehensive research.

## 1 Introduction

Heart failure (HF) and cognitive impairment are two common health conditions that often coexist in the general population (Virani et al., 2021). Both syndromes affecting cardiac and cognitive functions loom as public health problems in the coming decades due to the aging global population (Patnode et al., 2020; Díez-Villanueva et al., 2021).

HF is a clinical syndrome with symptoms and/or signs caused by a structural and/or functional cardiac abnormality affecting more than 60 million people worldwide (Groenewegen et al., 2020). Nearly half of the patients with HF are diagnosed with HF with preserved ejection fraction (HFpEF), a subclass of HF in which patients are more likely to be older and female, with multiple chronic comorbidities, such as hypertension, type 2 diabetes, and obesity (Owan et al., 2006; Dunlay et al., 2017). In addition to traditional comorbidities, cognitive impairment, which represents an independent risk factor for HF, significantly increases the hospitalization and mortality, and decreases quality of life of patients with HF (Dodson et al., 2013; Vellone et al., 2020).

Epidemiological evidence indicates that neurodegenerative processes share similar profiles with HF, in term of prevalence, age distribution, and mortality (Pappolla et al., 2003; Razay et al., 2007). Cognitive impairment encompasses a range of cognitive functions, including memory, attention, understanding, reasoning, problem-solving, decision-making, and language production (Murman, 2015). Interestingly, HF represents *per se* a risk factor for developing dementia (Qiu et al., 2006). Particularly prevalent in those over 70, where it reaches up to 10%, HF has long been recognized as a potential cause of cognitive dysfunction, sometimes referred to as cardiogenic dementia (Cardiogenic dementia, 1977; Liu et al., 2024). On the other hand, these syndromes can occur independently. The relationship between HF and cognitive impairment stems from partly common background. Age, metabolic disorders, genetic profile, or dysfunctional neuroendocrine systems are capable to affect both systems (Packer, 2018; Nguyen et al., 2020).

In this review, we will provide an overview of physiological connections between heart and brain. In particular, we will discuss the impact of metabolic disorders in affecting HF and cognitive function, and the implication of clinical treatments, focusing on paramount effects of new antidiabetic drugs.

## 2 A common milieu of metabolic disorders in heart failure and cognitive impairment

### 2.1 Heart failure

According to epidemiologic analyses, HF is highly prevalent in patients with type 2 diabetes, who are at a higher risk (up to 2-fold in men and 4-fold in women) of developing HF than patients without diabetes (Dunlay et al., 2019). However, it is important to note that type 2 diabetes, obesity, and metabolic dysfunction may specifically increase the likelihood of developing HFpEF more than HF with reduced ejection fraction (HFrEF) (Samson et al., 2016). Evidence from community-based cohorts has shown that higher body mass index and insulin resistance may be strongly associated with future HFpEF, especially among women (Savji et al., 2018). Furthermore, a sedentary lifestyle is associated with a greater risk of HFpEF compared with HFrEF (Pandey et al., 2016).

Several pathophysiological mechanisms linking type 2 diabetes and HF are recognized, with hyperglycemia, hyperinsulinemia, and insulin resistance known to initiate and perpetuate disease progression. Impairment in glucose metabolism, alterations of nitric oxide signaling, and reactive oxygen species (ROS) accumulation with mounting oxidative stress are among mechanisms underlying both micro- and macrovasculopathy, and diabetic cardiomyopathy with cardiac hypertrophy and fibrosis (Kolwicz and Tian, 2011; Piperi et al., 2015). Of note, insulin resistance is emerging as a major risk factor for the development of HF (Aroor et al., 2012a).

In this context, a prominent feature is functional alteration of mitochondria—the major source of intracellular ROS that compromise insulin signaling (Kim et al., 2008; Aroor et al., 2012b). In diabetic patients, ATP production relies even more on fatty acid oxidation rather than glycolysis (Lopaschuk, 2016). This process disrupts the oxidative phosphorylation and produces more ROS causing oxidative damage to cellular proteins and lipids (Gollmer et al., 2020). Changes in mitochondrial bioenergetics also affect myocardial ion handling resulting in cytosolic sodium and calcium overload (Trost et al., 2002; Bers, 2006). Abnormalities in the expression or activity of ryanodin receptor, sarcoplasmic endoplasmic reticulum Ca^2+^-ATPase, and sodium/calcium exchanger (NCX), reported in HF and diabetic cardiomyopathy, contribute to myocardial cell death, cardiac fibrosis, and diastolic dysfunction (Lebeche et al., 2008; Tuunanen and Knuuti, 2011).

In patients with type 2 diabetes, systemic chronic inflammation, caused by hyperglycemia and insulin resistance, participates in the development of vascular complications, and contributes to HF (Tsalamandris et al., 2019). Activation of nuclear factor-κB (NF-κB) results in downstream pro-inflammatory cytokine release, such as tumor necrosis factor-α (TNF-α), interleukin (IL)-6, and IL-1β, which amplify ROS-induced myocardial stress further promoting adverse remodeling, fibrosis, and myocardial dysfunction (Brasier, 2010; Jia et al., 2018).

The activation of renin-angiotensin-aldosterone system (RAAS) is associated with insulin resistance as well (Lastra et al., 2010). Upregulation of angiotensin II can activate NADPH oxidase enzyme complex in vascular smooth muscle cells and cardiomyocytes, further increasing the generation of ROS and participating in the progression of HF (Doughan et al., 2008). In addition, aldosterone, released upon the action of angiotensin II and sympathetic nervous system hyperactivation, reduces insulin sensitivity in several cell types triggering maladaptive responses in type 2 diabetes, hypertension, and HF (Aroor et al., 2012a). This is only a general outline, and excellent detailed reviews are available. However, the effective role of key pathways in the pathogenesis of cardiometabolic HF is still understudied and require further investigation. Understanding these mechanisms is crucial, as it can lead to the development of targeted interventions aimed at preserving cardiovascular health in individuals with type 2 diabetes.

### 2.2 Cognitive impairment

Cognitive impairment is generally referred to Alzheimer’s disease and vascular dementia, recognized as the two most prevalent causes of dementia that affects about 50 million people worldwide, with cases projected to triple by 2050 (GBD, 2016 Dementia Collaborators, 2019; Ou et al., 2024). Type 2 diabetes has increasingly represented a risk factor associated not only to cardiometabolic pathologies but also to neurological conditions, highlighting its negative impact on cognitive function (Banday et al., 2020; Tomic et al., 2022). Hyperglycemia in diabetic patients is a risk factor for Alzheimer’s disease that is also called “type 3 diabetes” (Barone et al., 2021).

The relationship between type 2 diabetes and cognitive impairment is multifaceted and intricate. Although exact mechanisms are still unknown, several potential pathways have emerged. The combination of vascular damage and neurodegeneration represents the main postulated mechanism underlying the development of cognitive impairment in patients with type 2 diabetes or HF (Dao et al., 2023). Chronic hyperglycemia and hyperinsulinemia drive a metabolic imbalance promoting oxidative stress, inflammation, and endothelial damage, responsible for non-enzymatic biomolecule glycation, accelerating cerebrovascular atherosclerosis and neurodegenerative damage that further promote cognitive dysfunction and dementia (Barone et al., 2021; Kopp et al., 2022). Under physiologic conditions, insulin promotes amyloid-β protein clearance thereby preventing its accumulation (Mullins et al., 2017). Alteration in insulin signaling may accelerate the onset of Alzheimer’s disease, by changing the intracellular concentration of neurotransmitters involved in memory formation and functioning, and influencing on amyloid-β-related processes and amyloid clearance (Craft, 2007; Spinelli et al., 2019; Rizzo et al., 2022). Additionally, insulin resistance increases the number of neuritic plaques by affecting tau phosphorylation, widely considered a pivotal pathological hallmark in Alzheimer’s disease (Wegmann et al., 2021).

Experimental studies provide strong evidence that insulin resistance-related neuroinflammation feeds cognitive impairment neuropathology, leading to a raised-up brain levels of pro-inflammatory cytokines (Mullins et al., 2017; Denver et al., 2018). Hyperphosphorylation of tau triggers pro-inflammatory signaling cascades, as a consequence of vascular inflammation and vasoconstriction (Paris et al., 2000). Activated microglia drive the immune response to the inflammatory state releasing immune signaling molecules (cytokines, and chemokines), and inducing neuronal cell death (Calsolaro and Edison, 2016).

These data underline the importance of optimal glycemic control and call for major pharmacological interventions to reduce the risk of cardiometabolic-related cognitive impairment.

**FIGURE 1:**
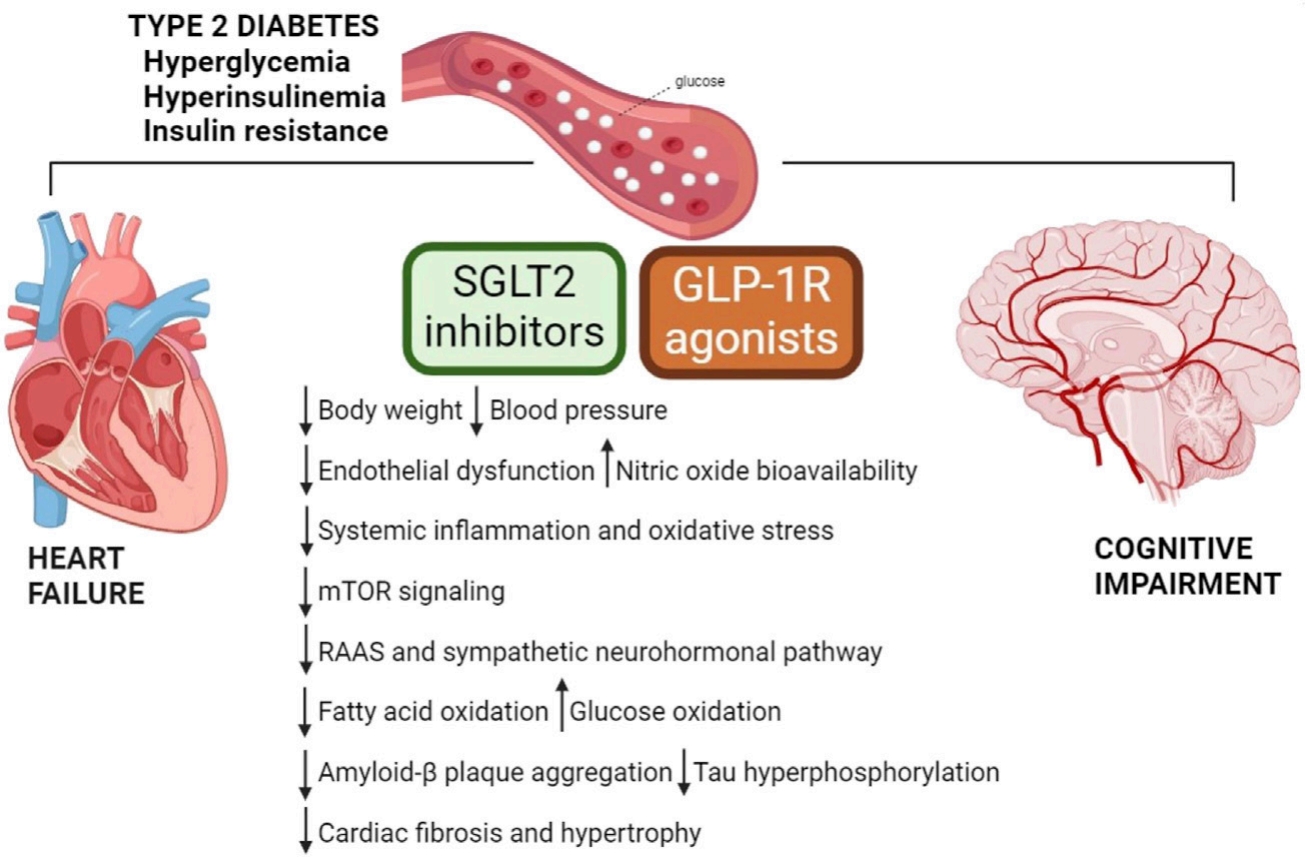
Mechanisms underlying favorable effects of GLP-1R agonists and SGLT2 inhibitors. Metabolic complications play a crucial role in the development and progression of HF and cognitive dysfunction. GLP-1R agonists and SGLT2 inhibitors exert direct and indirect cardioprotective and neuroprotective actions, and their benefits lay beyond glycemic control. GLP-1R, glucagon-like peptide-1 receptor; mTOR, mammalian target of rapamycin; RAAS, renin-angiotensin-aldosterone system; SGLT2, sodium-glucose cotransporter 2.

The pathophysiological overlap between cardiometabolic HF and cognitive impairment opens to reasonable possibility that new successful pharmacological approaches used in type 2 diabetes may be beneficial for cardiovascular and neurological diseases. Indeed, there is growing interest in the potential of new classes of anti-diabetic medications such as glucagon-like peptide-1 receptor (GLP-1R) agonists and sodium-glucose cotransporter 2 (SGLT2) inhibitors, to reduce the risk of cardiovascular events and slow down the progression of neuronal damage. Notably, these drugs not only act on the glycemic control, but also show protective effects independently of their anti-diabetic effect (Table 1, Figure 1).

**TABLE 1:** Summary of cardioprotective and neuroprotective mechanisms by GLP-1R agonists and SGLT2 inhibitors.

Site of action	GLP-1R agonists	SGLT2 inhibitors
**Heart**	Increasing of atrial natriuretic peptide and nitric oxide, reduction of blood pressure (Kim et al., 2013; Richards et al., 2014; Drucker, 2016; Baggio et al., 2018; Pandey et al., 2023)	Increasing of diuresis and natriuresis, reduction of blood pressure (Mazidi et al., 2017; Dominguez Rieg and Rieg, 2019)
	Suppression of oxidative stress (Drucker, 2016; Helmstädter et al., 2020; Baylan et al., 2022)	Suppression of oxidative stress (Frati et al., 2017; Checa and Aran, 2020; Chen et al., 2022; Elrakaybi et al., 2022)
	Inhibition of pro-inflammatory cytokines, NLRP3 inflammasome, promotion of M2 macrophage phenotype (Hogan et al., 2014; Bułdak et al., 2016; Bruen et al., 2017; Luo et al., 2019; Yu et al., 2019; Püschel et al., 2022)	Inhibition of pro-inflammatory cytokines, NLRP3 inflammasome, promotion of M2 macrophage phenotype (Lee et al., 2017; Ye et al., 2017; Byrne et al., 2020; Kim et al., 2020)
	Energetic shift from fatty acid to glucose oxidation (Bao et al., 2011)	Modulation of myocardial hypertrophy and fibrosis (Bay et al., 2013; Croteau et al., 2021)
	Activation of SIRT1 signaling (Luo et al., 2019)	Regulation of sodium and calcium homeostasis (Cappetta et al., 2020; Uthman et al., 2022)
	Inhibition of RAAS pathway (Kim et al., 2013; Jensen et al., 2020; Martins et al., 2020)
		Inhibition of RAAS and sympathetic neurohormonal pathways (Salvatore et al., 2022)
**Brain**	Reduction of neuroinflammation, decrease of microglia activation, promotion of M2 macrophage phenotype (Hogan et al., 2014; Bułdak et al., 2016; Bruen et al., 2017; Yu et al., 2019; Zhang et al., 2021a; Püschel et al., 2022)	Reduction of NLRP3 inflammasome, decrease of microglia activation, promotion of M2 macrophage phenotype (Lin et al., 2014; Iannantuoni et al., 2019; Xu et al., 2019; Lee et al., 2020; Lonnemann et al., 2020; Lee et al., 2021; Khedr et al., 2024)
	Reduction of amyloid-β plaque deposition and tau hyperphosphorylation (Li et al., 2012; Long-Smith et al., 2013; McClean and Hölscher, 2014; McClean et al., 2015; Cai et al., 2018)	Reduction of amyloid-β plaque deposition and tau hyperphosphorylation (Huang et al., 2016; Cenini and Voos, 2019; Lonnemann et al., 2020)
	Activation of brain-derived neurotrophic factor (Du et al., 2022)	Activition of brain-derived neurotrophic factor (Arab et al., 2021)
	Enhancement glucose metabolism (Bomba et al., 2013; Zheng et al., 2021)	Interference with mTOR signaling (Packer, 2020; Stanciu et al., 2021; Samman et al., 2023)

## 3 Glucagon-like peptide-1 receptor agonists

Glucagon-like peptide-1 (GLP-1) represents a particular incretin that has generated a lot of interest. Discovered for the first time in 1987, GLP-1 is an intestinal gut hormone that is secreted in response to food intake and potentiates the glucose-dependent insulin secretion from pancreatic β-cells (Drucker et al., 1987). It acts by binding to the G protein-coupled receptor GLP-1 receptor (GLP-1R). In addition to metabolic effects (promotion of insulin secretion, inhibition of appetite, reduction of gastric emptying, and increase of natriuretic and diuretic processes), experimental studies have pointed out the cardio- and neuroprotective effects of GLP-1 (During et al., 2003; Bendotti et al., 2022; Yang et al., 2022). GLP-1 is a labile peptide and is rapidly removed from circulation by enzymatic degradation by the serine protease dipeptidyl peptidase (DPP)-4. The therapeutic approach to target the incretin system for the treatment of type 2 diabetes involves the use of GLP-1 agonists or DPP-4 inhibitors to increase the levels of endogenous GLP-1 (Cosentino et al., 2020).

In addition to pancreatic localization, GLP-1R has been found in the gastrointestinal system, kidneys, central nervous system, and cardiovascular system (Pyke et al., 2014). The additional non-glycemic effect related to incretins may be linked to extra-pancreatic tissue localization of GLP-1R.

GLP-1R agonists are classified on their molecular backbone derived either from human GLP-1 (liraglutide, semaglutide, and dulaglutide) or from exendin-4 (exenatide and lixisenatide), a salivary gland hormone from the Gila monster lizard (Colin et al., 2023). In addition, a dual GLP-1/glucose-dependent insulinotropic polypeptide (GIP) co-agonist, tirzepatide, has been recently developed for the treatment of type 2 diabetes and obesity (Jakubowska et al., 2024). GIP is an incretin hormone secreted by small intestine in response to luminal presence of nutrients (Deacon and Ahrén, 2011). It stimulates insulin secretion, facilitates fat deposition, promotes bone formation and may be involved in memory formation and control of appetite (Seino et al., 2010). Changes in GIP secretion could contribute to the loss of incretin effect observed in patients with type 2 diabetes (Holst et al., 2011).

GLP-1R agonists are known to significantly decrease the cardiovascular risk of diabetic patients, while they are currently being investigated to determine their potential efficacy for neurodegenerative diseases associated to cardiometabolic pathology.

### 3.1 GLP-1R agonists in the cardiovascular system

GLP-1R is expressed in the heart and vasculature, raising the possibility that GLP-1 might have both direct and indirect actions on the cardiovascular system (Ussher and Drucker, 2023). GLP-1R expression has been found in both the atria and ventricles (Baggio et al., 2018). Its regulation contributes to the reduction of blood pressure through the secretion of atrial natriuretic peptide or by regulating the availability of nitric oxide (Pandey et al., 2023). GLP-1 and its receptor agonists have been shown to reduce endothelial inflammation and oxidative stress, through the activation of nitric oxide production, crucial for maintaining healthy vascular tone and function (Drucker, 2016).

Incretin-based therapies has evidenced a reduced cardiovascular risk in high-risk individuals with type 2 diabetes, independent of the need for additional glucose control. Among GLP-1R agonists, liraglutide, semaglutide, and dulaglutide are widely available with licensed indications for the prevention of cardiovascular disease (Marx et al., 2022).

However, understanding how activation of the GLP-1R contributes to myocardial protection and decreases cardiac injury is challenging.

GLP-1R activation reduces blood pressure, as shown in experimental models of hypertension (Hirata et al., 2009; Laugero et al., 2009). The anti-hypertensive effect of GLP-1R agonists has been observed with liraglutide that is able to mediate atrial natriuretic peptide secretion from atrial cardiomyocytes, thus lowering systolic and diastolic blood pressure increased by angiotensin II (Kim et al., 2013). In addition, it has been demonstrated the specific involvement of endothelial GLP-1R in contributing to the anti-hypertensive effects by controlling nitric oxide bioavailability (Richards et al., 2014; Baggio et al., 2018). Liraglutide shows efficacy in experimental arterial hypertension, independently of glycemic control. Mechanistically, liraglutide suppresses the angiotensin II-induced inflammatory cascade and oxidative stress in the vascular wall, leading to reduced expression of adhesion molecules at endothelial level. As a result, function of endothelial nitric oxide synthase is preserved, thus restoring nitric oxide level (Helmstädter et al., 2020; Baylan et al., 2022). Additionally, the relevant effect of GLP-1R signaling on blood pressure control may be attributable to the action of agonists on sodium reabsorption in the proximal tubule, and interference with intrarenal angiotensin II, thus augmenting renal blood flow and promoting natriuresis (Jensen et al., 2020; Martins et al., 2020).

Reduced cardiac and vascular inflammation has been well documented for GLP-1R agonists in experimental and human studies (Drucker, 2016). Liraglutide lowers vascular dysfunction and systemic inflammation in a lipopolysaccharide-induced sepsis model, by normalizing the expression of adhesion molecules and cytokines (Steven et al., 2015). Interestingly, in GLP-1R-deficient mice, liraglutide loses its beneficial effects (Steven et al., 2017). In regulating the response to inflammation of the endothelium, dulaglutide counteracts high glucose-induced activation of the NLR family pyrin domain containing 3 (NLRP3) inflammasome and suppresses maturation and release of IL-1β and IL-18; the lack of effects of dulaglutide on endothelial inflammation by SIRT1 siRNA evidences the involvement of SIRT1 pathway in this process (Luo et al., 2019). In human peripheral blood cells, liraglutide has been reported to decrease the production of pro-inflammatory cytokines (TNF-α, IL-1β, and IL-6), and activation of macrophages (Hogan et al., 2014; Bruen et al., 2017). Under pathological stress, the role of macrophages in inflammatory responses, which drive insulin resistance and diabetes, is significant (Püschel et al., 2022). In this regard, GLP-1R agonists (i.e., exenatide) improve insulin resistance suppressing pro-inflammatory phenotypes of monocytes/macrophages, and directly modulating NF-κB activation and IL-1β, IL-6, and TNF-α expression (Bułdak et al., 2016; Yu et al., 2019).

Data suggests that GLP-1R-dependent cardioprotection may also result from effects on cardiac energetic metabolism. In ischemia–reperfusion model, albiglutide promotes energetic shift from fatty acid to glucose oxidation, reducing myocardial infarct size and improving contractile function (Bao et al., 2011). Likewise, liraglutide increases myocardial glucose oxidation, and alleviates diastolic dysfunction in mice subjected to experimental diabetic cardiomyopathy (Almutairi et al., 2021).

Dual GLP-1R/GIP receptor agonists co-stimulate both GLP-1R and GIP receptor, and enhance insulinotropic and antihyperglycemic effects compared with selective GLP-1R agonists (Finan et al., 2013). Tirzepatide is the only approved incretin multi-agonist. It has been proven superior to single GLP-1R agonist or insulin analog treatments in reducing blood glucose, body mass index, and weight (Dutta et al., 2021). Tirzepatide has been associated with improvements in lipoprotein profiles, blood pressure, and inflammation, although favorable effects on cardiovascular risk factors have been reported in only a single clinical trial (Wilson et al., 2020; Del et al., 2021).

Triple agonists are unimolecular peptide drugs that target GLP-1R, GIP receptor, and glucagone receptor, developed primarily for treating metabolic diseases (Knerr et al., 2022). Preclinical data available so far have highlighted metabolic efficacy and its superior performance to dual agonists in reducing cardiovascular risk factors, such as plasma glucose levels, and body weight (Finan et al., 2015; Knerr et al., 2022).

The above findings underscore the potential of GLP-1R agonists as efficacious and safe drugs with pleiotropic activities on cardiovascular system that are independent of their glycemic control effects emphasizing cardiovascular outcomes as one of the primary goals in the management of type 2 diabetes.

### 3.2 GLP-1R agonists in the central nervous system

GLP-1R, expressed in endothelial cells, microglia, astrocytes, and neurons, is widely distributed in the hypothalamus, cortex, subventricular zone, and substantia nigra (Hamilton and Holscher, 2009; Cork et al., 2015; Reich and Hölscher, 2022). GLP-1R overexpression has been found in neurons, microglia, and endothelial cells, where is correlated to low level of inflammation and improved cognitive function (Du et al., 2022). GLP-1R activation, by stimulating adenylyl cyclase and consequently elevating cyclic adenosine monophosphate levels, enhance neuroprotective signaling through upregulation of anti-inflammatory and anti-apoptotic signaling (Holz, 2004; Delghandi et al., 2005; Robichaux and Cheng, 2018; Wicinski et al., 2019; Mehdi et al., 2023).

Pharmacological approaches able to promote healthy incretin and insulin signaling in the brain may represent a potential direction for neurodegenerative disease treatment. Clinical trials have pointed out a neuroprotective effect and an improvement of clinical outcome for some anti-diabetic treatments, translating them into new therapies against neurodegenerative conditions, such as Alzheimer’s disease, dementia, or Parkinson’s disease (Green et al., 2019; Kopp et al., 2022; Colin et al., 2023).

GLP-1R agonists cross the blood-brain barrier and have been widely tested in preclinical studies as therapeutic option for neuroinflammation and neurodegeneration. In an experimental model of Alzheimer’s disease, liraglutide exerts neuronal anti-inflammatory effects, decreasing the number of activated microglia cells in the hippocampus, and astrocyte in the cortex as well as reducing the expression of pro-inflammatory cytokines (IL-6, IL-12, and IL-1β). It also shows properties as growth and differentiation factor, increasing stem cell proliferation and differentiation into mature neurons (Parthsarathy and Holscher, 2013; Batista et al., 2018). In a model of cognitive impairment associated to neuropathic pain, the inhibition of GLP-1/GLP-R axis increases the expression of pro-inflammatory cytokines IL-1β and TNF-α, while exenatide treatment prevents the phosphorylation of NF-κB and decreases inflammatory burden (Zhang LQ. et al., 2021).

GLP-1 activation can reduce the pathological process of amyloid-β plaque aggregation/deposition and tau hyperphosphorylation enhanced by neuroinflammation, thus preventing age-related neurodegenerative changes, such as decline of learning and memory (Li et al., 2012). Lixisenatide is a long-lasting GLP-1R agonist that crosses blood-brain barrier at very low doses (Hunter and Holscher, 2012). In a mouse model of Alzheimer’s disease, it reduces amyloid-β plaques, tau neurofibrillary tangles, and neuroinflammation, by activating CREB pathway and inhibiting p38/MAPK signaling (Cai et al., 2018). A large body of evidence reports a beneficial effect of liraglutide on amyloid pathology. The drug, as prophylactic or long-term treatments, significantly reduces amyloid-β plaque size, number, and load (Long-Smith et al., 2013; McClean and Hölscher, 2014; McClean et al., 2015). Both liraglutide and dulaglutide ameliorate impaired learning and memory ability limiting tau hyperphosphorylation and neurofilament aggregates through the modulation of JNK or Akt activity (Qi et al., 2016; Chen et al., 2017; Zhou et al., 2019).

Neuronal apoptosis, induced by plaque deposition and stress, is a pathophysiological marker in cognitive impairment (Engidawork et al., 2001). In a mouse model of Parkinson’s disease, both lixisenatide and liraglitude are associated with neuroprotective benefits and reduced motor impairment by decreasing pro-apoptotic BAX signaling and increasing expression of anti-apoptotic Bcl-2 (Liu et al., 2015). Likewise, the neuroprotective effect of semaglutide involves modulation of apoptotic signaling (Chang et al., 2020).

GLP-1R agonists may also exert their neuroprotective action increasing level and activity of brain-derived neurotrophic factor, regulating calcium homeostasis, promoting glycolysis, and reducing vascular damage (Du et al., 2022). Of note, it has been shown that liraglutide improves learning and memory as well as cognitive function by regulating intracellular calcium homeostasis in cortical or hippocampal pyramidal cells (Wang et al., 2013). Moreover, both exenatide and liraglutide enhance glucose metabolism and aerobic glycolysis by increasing brain lactate dehydrogenase activity (Bomba et al., 2013; Zheng et al., 2021).

Dual GLP-1R/GIP receptor agonists exert anti-neuroinflammatory and anti-neurodegenerative benefits in preclinical studies and improve memory function, synaptic health, and neurogenesis (Pathak et al., 2018; Zhang et al., 2020). Interestingly, the dual agonist tirzepatide has shown efficacy in neuroprotection, by counteracting hyperglycemia and insulin resistance-related effects at the neuronal level (Fontanella et al., 2024).

Also triple agonists have demonstrated to possess anti-neurodegenerative properties, indicating a potential for treating Alzheimer’s disease by reducing neuroinflammation and oxidative stress, enhancing neuronal health and viability, and minimizing glutamate excitotoxicity (Kopp et al., 2022). In mouse models of Alzheimer’s disease, triple agonists play a neuroprotective role and ameliorate cognitive deficits by significantly reducing hippocampal amyloid-β, phosphorylated tau aggregates, and neuroinflammation, and stimulating neurogenesis and brain-derived neurotrophic factor expression (Li et al., 2018; Tai et al., 2018).

All these experimental data support the potential benefit of this drug class to translate neuroprotective properties from cellular and animal studies to humans. Although several clinical trials have been completed or are currently underway, repurposing GLP-1R agonists to treat cardiometabolic syndrome-related neurodegenerative diseases will require additional research to confirm safety and efficacy.

## 4 Sodium/glucose co-transporter 2 inhibitors

Sodium/glucose co-transporter 2 (SGLT2) inhibitors or gliflozins represent a new class of approved oral anti-hyperglycemic drugs widely used in clinical practice for the treatment of type 2 diabetes, alone or in association with metformin (Kalra, 2014; Nauck, 2014). Their main mechanism of action consists in the inhibition of SGLT receptors, of which two different isoforms are known, SGLT1 and SGLT2. SGLTs are membrane proteins that act as co-transporters of glucose and sodium in the cell, and their expression and function have been detected in many organs of the body (Pandey and Tamrakar, 2019; Koepsell, 2020). Selective SGLT2 inhibitors block glucose reabsorption in the proximal renal tubule, increasing glucose elimination and reducing hyperglycemia. They improve both insulin secretion by β-cells and peripheral insulin sensitivity (Ni et al., 2020).

Dapagliflozin, empagliflozin, and ertugliflozin are the most selective SGLT2 inhibitors (Ferrannini, 2017). Sotagliflozin, referred to as a dual SGLT1/2 inhibitor, has the highest affinity for SGLT1 (Markham and Keam, 2019), while canagliflozin can act as a dual SGLT1/2 inhibitor and is able to inhibit intestinal glucose absorption by blocking intestinal SGLT1 (Dominguez Rieg and Rieg, 2019). Although these drugs were initially developed for the treatment of type 2 diabetes, clinical studies have largely demonstrated their beneficial effects in metabolic, renal, and cardiovascular diseases that go beyond glucose control (Minze et al., 2018; Mascolo et al., 2022a; Urbanek et al., 2023).

Based on the results of clinical data, empagliflozin and dapagliflozin are now recommended for the treatment of HFpEF, as well as for HFrEF also in the absence of diabetes (McDonagh et al., 2021; McDonagh et al., 2023).

### 4.1 SGLT2 inhibitors in the cardiovascular system

Numerous studies have proved that SGLT2 inhibitors reduce the combined risk of cardiovascular death and hospitalization in HF patients with or without diabetes. The benefits include osmotic diuresis and natriuresis, weight loss and lipid metabolism shift, blood pressure reduction, decrease in uric acid, oxidative stress, and inflammation with a lower risk of hypoglycaemia or pancreatic β-cell overstimulation. They represent a new therapeutic approach able to act directly on the heart or kidneys, independently of insulin sensitivity (Lopaschuk and Verma, 2020; Goldberg, 2021). SGLT2 inhibitor effects have been established on cardiomyocytes, endothelial cells, fibroblasts, and smooth muscle cells (Uthman et al., 2018a; Cappetta et al., 2021).

Oxidative stress and inflammation significantly and interdependently contribute to the onset and progression of cardiovascular diseases (Frati et al., 2017). Inflammation can induce acute or chronic oxidative stress via cytosolic protein kinase C and calcium release; *vice versa*, oxidative stress is a positive modulator of the inflammatory response through NLRP3 inflammasome and NF-κB (Checa and Aran, 2020; Chen et al., 2022). SGLT2 inhibitors exhibit considerable potential as anti-inflammatory agents, through indirect mechanisms that involve metabolic improvement, oxidative stress reduction, as well as direct modulation of inflammatory pathways (Elrakaybi et al., 2022). In obese mice with diabetes, dapagliflozin reduces cardiac dysfunction interfering with activation of NLRP3 inflammasome and affects cardiac fibrosis by attenuating collagen 1 and collagen 3 deposition (Ye et al., 2017). Similarly, empagliflozin suppresses NLRP3 inflammasome activation in macrophages of diabetic patients (Kim et al., 2020). Furthermore, it has been shown that empagliflozin attenuates the decline of cardiac function in mice with HF in the absence of diabetes and that this is associated with reduced priming of NLRP3 inflammasome in the heart (Byrne et al., 2020). In post-ischemic rat hearts, treatment with dapagliflozin results in higher level of IL-10, a cytokine that facilitates the conversion of macrophages from a pro-inflammatory M1 to anti-inflammatory M2 phenotype, thereby inhibiting myofibroblast differentiation and extracellular matrix formation (Lee et al., 2017).

Cardiomyocytes and endothelial cells are major targets for SGLT2 inhibitors in the heart (Chen et al., 2022). Studies support that intracellular sodium and calcium overload induce signaling cascades that lead to dysregulation of mitochondrial homeostasis, with energy impairment and elevated free radical production, and consequent increase in cardiac hypertrophy and remodeling (Bay et al., 2013). In mice with diabetic cardiomyopathy, ertugliflozin prevents myocardial hypertrophy, fibrosis, and diastolic dysfunction by preserving mitochondrial function and improving myocardial energetics (Croteau et al., 2021). Activation of NCX and sodium/hydrogen exchanger-1 (NHE-1), at cardiac and vascular level, mediate the maladaptive neurohormonal stimulation (sympathetic nervous system, RAAS, and natriuretic peptide system) during the pathophysiological course toward HF (Baartscheer et al., 2003; Packer, 2017). SGLT2 inhibitors show a strict interaction with human and murine cardiomyocytes through direct inhibition of the myocardial isoform of NHE (Uthman et al., 2018b; Trum et al., 2020). Moreover, both dapagliflozin and empagliflozin have been shown to directly inhibit NHE-1 activity in human endothelial cells (Cappetta et al., 2020; Uthman et al., 2022). Another factor in the development of HF is the late component of the cardiac voltage-gated sodium current, termed late-INa (Horvath and Bers, 2014). *In silico* approach confirms a reduction of late-INa by empagliflozin demonstrating that SGLT2 inhibitors may be the ligands for the cardiac sodium channel Nav1.5 isoform that drives late-INa (Philippaert et al., 2021). Amelioration of dysfunctional sodium and calcium homeostasis in cardiomyocytes from diabetic hearts is able to reverse cardiac remodeling and counteract the development of diabetic cardiomyopathy (Lee et al., 2019; Philippaert et al., 2021). Moreover, the regulation of sodium and calcium homeostasis in cardiomyocytes and coronary endothelium is directly translated into improved contractility and relaxation of the heart (Cappetta et al., 2020).

Diuretic and natriuretic effects of SGLT2 inhibitors contribute to the reduction of intravascular volume and decrease in blood pressure, lowering afterload, improving cardiac efficiency, and reducing ventricular mass index (Mazidi et al., 2017; Dominguez Rieg and Rieg, 2019). SGLT2 inhibitors can reduce natriuretic peptides leading to a reduction in ventricular pressure and distention, and both pulmonary and systemic decongestion (Mascolo et al., 2022b). Moreover, the favorable hemodynamic effects seem to be related with an attenuated endothelial cell activation and improved vasorelaxation *via* voltage-gated potassium channels (Dimitriadis et al., 2023). Additionally, benefits include the capacity to decrease the RAAS and sympathetic neurohormonal pathways and a positive pharmacodynamic interaction with RAAS inhibitors (Salvatore et al., 2022).

The above cardiac and extracardiac effects outline the cardioprotective mechanisms of SGLT2 inhibitors that stay behind the success of these drugs in the cardiovascular field.

### 4.2 SGLT2 inhibitors in the central nervous system

SGLT1 is localized in the pyramidal cells of the brain cortex, Purkinje cerebellum cells, hippocampus pyramidal, and granular cells (Pawlos et al., 2021). SGLT2 brain expression is lower compared to SGLT1, and it is localized mainly in the microvessels of the blood-brain barrier, in the amygdala, hypothalamus, periaqueductal gray, and in the dorsomedial medulla (Enerson and Drewes, 2006). Both co-transporters are also described in the membrane of the capillary endothelium (Koepsell, 2020). Interestingly, the presence of SGLT1/2 co-transporters has been found mainly in areas involved in processes such as learning, food intake, energy and glucose homeostasis, and central cardiovascular and autonomic regulation (Wiciński et al., 2020).

Growing evidence suggests that SGLT2 inhibitors have a neuroprotective potential and may improve the impaired cognitive function of diabetic patients by exerting pleiotropic effects and modulating numerous molecular mechanisms.

Chronic inflammation negatively affects the blood-brain barrier and leads to a pro-inflammatory phenotype of astrocytes and microglia (Rochfort and Cummins, 2015). Pro-inflammatory M1 polarization associates with neurodegeneration and cognitive impairment (Zhang Z. et al., 2021). SGLT2 inhibitors (i.e., dapagliflozin) have been proven to strongly promote macrophage polarization towards a M2 phenotype, thus alleviating inflammation and atherosclerotic processes (Lee et al., 2020). Similarly, empagliflozin can mitigate inflammation and macrophage stimulation by downregulating JAK2/STAT1 pathway and optimize energy expenditure (Xu et al., 2019; Lee et al., 2021). SGLT2 inhibitors may also regulate macrophage activation by decreasing the generation of free radicals and enhancing the expression of antioxidant enzymes (Lin et al., 2014; Iannantuoni et al., 2019). In Alzheimer’s disease, NLRP3 inflammasome has emerged as a pathological component participating in the impaired removal of amyloid-β by microglia (Lonnemann et al., 2020). Notably, SGLT2 inhibitors empagliflozin and canagliflozin act on macrophages and attenuate NLRP3 inflammasome in atherosclerosis and cognitive impairment, respectively (Kim et al., 2020; Khedr et al., 2024).

The inflammatory state may contribute to the overproduction of ROS or the decrease in antioxidant defense by compromising mitochondrial function (Guo et al., 2013). Oxidative stress is associated with amyloid-β- or tau-induced neurotoxicity, which results in impaired synaptic plasticity, neurotransmitter imbalance, and neuronal loss, leading to cognitive impairment (Huang et al., 2016; Cenini and Voos, 2019). Interestingly, the administration of dapagliflozin in obese mice is associated with a significant improvement in brain mitochondrial function and decreased ROS production, preventing cognitive decline (Sa-Nguanmoo et al., 2017).

Another crucial target involved in metabolic diseases and cognitive impairment is the mammalian target of rapamycin (mTOR). Hyperglycemia-induced mTOR upregulation leads to blood-brain barrier disruption and endothelial dysfunction, and contributes to tau hyperphosphorylation and amyloid-β aggregation in Alzheimer’s disease (Van Skike and Galvan, 2018; Uddin et al., 2020). SGLT2 inhibitors contribute to switching energy metabolism and reinstate mTOR activity in cardiovascular system, and by restoring mTOR cycle may interfere with a cognitive impairment (Packer, 2020; Stanciu et al., 2021).

Brain insulin resistance is heavily implicated in the pathogenesis of Alzheimer’s disease, and takes part in amyloid-β- and tau phosphorylation-mediated neuronal damage (Mullins et al., 2017). In a murine model of Alzheimer’s disease and type 2 diabetes, empagliflozin has been shown to ameliorate metabolic alterations and glucose levels lowering senile plaque burden; these effects are accompanied by an improvement of cognitive deficits (Hierro-Bujalance et al., 2020). In an experimental model of Alzheimer’s disease induced by aluminum chloride, dapagliflozin shows neuroprotective effects by suppressing brain amyloid-β protein deposition; improved cognition is mediated through different mechanisms, such as attenuation of oxidative stress, enhancement of glucose metabolism, and regulation of mTOR signaling (Samman et al., 2023). Moreover, dapagliflozin markedly alleviates neuronal oxidative stress, tau phosphorylation and amyloid-β production due to the ability to counteract neuronal apoptosis and upregulating glial cell-derived neurotrophic factor (Arab et al., 2021).

Alzheimer’s disease is also characterized by a reduced level of neuronal acetylcholine and the first line pharmacological treatment is based on the use of acetylcholinesterase inhibitors (Ferreira-Vieira et al., 2016). In an experimental model of cognitive impairment induced by scopolamine, the beneficial effect of canagliflozin on cognitive function has been attributed to the capacity to inhibit acetylcholinesterase and increase acetylcholine M1 receptor expression (Arafa et al., 2017). These results have been confirmed by molecular docking studies evaluating the binding energy of dapagliflozin to the catalytic site of SGLT2 and acetylcholinesterase enzymes, thus suggesting that dapagliflozin might act as a dual inhibitor to treat diabetes-associated neurological disorders (Shaikh et al., 2016).

## 5 Future directions

The mechanisms underlying HF and cognitive impairment have been extensively investigated although studies to provide a comprehensive view of the pathophysiological aspects at stake are needed. At this regard, epigenetic signature, observed in type 2 diabetes as well as in chronic cardiovascular and neurological diseases, is emerging as an innovative research field and a potential pharmacological target. Future research should explores the capability of GLP-1R agonists and SGLT2 inhibitors to modulate epigenetic writers, readers and erasers in the context of cardiovascular and brain homeostasis (Scisciola et al., 2020; Donniacuo et al., 2023).

The effectiveness of these anti-diabetic drugs in HF patients has been clearly established. Instead, the use of GLP-1R agonists and SGLT2 inhibitors as a potential therapeutic option for cognitive dysfunction is a relatively new area of investigation, and clinical studies are still limited. Therefore, more research is needed to understand the underlying mechanisms and to determine long-term safety and efficacy in humans. Intriguingly, the association of these pharmacological classes may represent a promising therapeutic strategy, and some clinical studies have already shown adjunctive benefits of the combination therapy.

Given the impact of type 2 diabetes on cardiovascular and neurological diseases, GLP-1 agonists and SGLT2 inhibitors represent a valid pharmacological tool for improving cardiometabolic profile and diabetes-related cognitive impairment. Because the vast array of molecular and cellular mechanisms modulated by these two new drug classes often intercept in pathophysiological background of chronic cardiovascular and neurodegenerative diseases, it is possible that any future new anti-diabetic drug may be also relevant for cardiometabolic and cognitive decline, independently from the anti-diabetic action.

## Abbreviations

DPP-4, protease dipeptidyl peptidase 4; GIP, glucose-dependent insulinotropic polypeptide; GLP-1, glucagon-like peptide-1; GLP-1R, glucagon-like peptide-1 receptor; HF, heart failure; HFpEF, heart failure with preserved ejection fraction; HFrEF, heart failure with reduced ejection fraction; IL, interleukin; INa, sodium current; mTOR, mammalian target of rapamycin; NCX, sodium/calcium exchanger; NF-κB, nuclear factor-κB; **NHE-1**, sodium/hydrogen exchanger-1; **NLRP3**, NLR family pyrin domain containing 3 inflammasome; **RAAS**, renin-angiotensin-aldosterone system; **ROS**, reactive oxygen species; **SGLT2**, sodium-glucose cotransporter 2; **TNF-α**, tumor necrosis factor-α.
